# Seeking exposure: conversions of scientific knowledge in an African city*

**DOI:** 10.1017/S0022278X16000240

**Published:** 2016-09

**Authors:** Gemma Aellah, P. Wenzel Geissler

**Affiliations:** London School of Hygiene and Tropical Medicine, Keppel Street, London WC1E 7HT, United Kingdom and The Royal Anthropological Institute, 50 Fitzroy Street, London W1T 5BT, United Kingdom; Department of Social Anthropology, University of Oslo, Norway and Department of Archaeology and Anthropology, University of Cambridge, Cambridge, United Kingdom

## Abstract

Transnational medical research has become a common feature in many parts of Africa. This paper explores the contribution such activity makes to the social and economic lives of those involved, including both trial subjects and local staff. By considering the value of the ‘exposure’ that involvement brings to staff and research participants, we reflect on the conversion of scientific knowledge into practical knowledge and its value to sustaining precarious livelihoods in an economically fragile city. We consider the interplay between science and sociality and argue for a need to take seriously the circulation of scientific knowledge beyond the confines of expert spaces.

## INTRODUCTION

December 2009. In an African city two women prepare to leave their houses for the day. Emily,^1^ a single mother and nurse working at a transnational medical research centre, packs the second-hand clothes she bought at the market and is planning to resell to her colleagues. She says goodbye to her daughters, reminding her house-help that she will return late because she wants to use the office computer to study for an online diploma course. Emily leaves her rental flat above a shop, taking her employment ID card. Avoiding rubbish strewn across the road, she boards a public minibus. The blaring bus radio promotes male circumcision as HIV prevention, reminding listeners that this city has the highest HIV prevalence in the country.

Meanwhile, in her mud-walled bedsit, Jennipher, a research participant in an HIV prevention trial, prepares breakfast for her husband, who does roadside radio repairs. The charcoal briquettes she makes from coal dust and clay to sell to her neighbours are piled in the corner. Jennipher packs a bag with a cloth and flask of porridge for her toddler who is freshly bathed, powdered and wearing a frilly dress. Today Jennipher is taking her daughter to the research centre for their final visit. She plans to ask the doctor to write her a recommendation letter for a peer-education position she has seen advertised at an HIV Treatment Centre. Jennipher did not complete school and hopes a note from the doctor describing her knowledge about HIV, acquired through her trial participation, will suffice. She grabs her research participant ID card and walks to the centre. Roadside billboards advise her to carry a condom, call abroad from 10 bob a minute and invest in shares. She passes signposts for various HIV-related NGOs and tents used for mobile HIV testing. She enters the ‘Obama Gate’ to the public hospital compound. There she passes Emily, the study nurse, alighting from her minibus. They catch each other's eye and smile, as both display their ID cards to gain entrance to the guarded research centre within.

Emily and Jennipher live, work and plan for their families' futures in uncertain situations in an African city. In doing so, they both engage with scientific knowledge involved in the practices of HIV/AIDS research. One is a research participant in a trial to reduce mother-to-child transmission of HIV conducted by a parastatal National Clinical Research Organisation (NCRO) in collaboration with a Central Health Agency (CHA) from the Global North.^2^ The other is a former government nurse working for the same trial, forging a new career in medical research. Drawing upon the narratives of research staff and participants like these, and ethnographic observations of HIV research work over several years, this paper explores the, sometimes unexpected, movement of scientific knowledge around a ‘trial community’ (Geissler 2011) and its relation to the identities, imagination and ambitions of people involved.

Many parts of Africa are experiencing the profound impact of large-scale biomedical research programmes, intertwined with much larger transnational HIV/AIDS interventions (Crane 2013; Prince & Marsland 2013; Geissler 2015), both premised on a broadly ‘experimental’ logic, generating knowledge as they go along (e.g. Nguyen 2009; Rottenburg 2009). By focusing on the production and use of scientific knowledge, our paper aims to reflect on the interplay between science and sociality in an African city today, contributing both to our understanding of post-colonial science and the conditions of precariousness that shape 21st century urban life across the continent (Cooper & Pratten 2014). We draw on a metaphor of ‘exposure’, which emerged directly from the narratives of our interlocutors, to explore the movement and remaking of scientific knowledge in this exceptional, but not singular, space of hyper-medicalised development. Using this fertile metaphor, we follow the energetic radiation of knowledge through, outwards and back to the ‘trial community’, knowledge that often changes on its journeys, sometimes doing good, sometimes not, but always intensely productive.

### Scientific knowledge in a researched city

We use ‘scientific knowledge’ as shorthand for knowledge that results from scientific work in a given place, and that retains connections or reference to its scientific origins. We are not focusing here on the abstraction of ‘Science’ as in the making and circulation of scientific facts in a global scientific community. Anthropology already has a strong tradition of prising open the sociality of making scientific facts (Latour & Woolgar 1979). Instead, we look at a more obviously localized circulation of knowledge; the epistemological ‘spillage’ or ‘radiation’ of scientific work itself. This may include ‘proper’ scientific end products like statistics and publications, but much of it consists of practical knowledge created through production processes: technical procedures, habits and behaviours as well as social strategies, modes of self-presentation and of relating to co-workers and others. It also includes career ambitions and lifestyles. Such knowledge is not necessarily aimed for, nor always formalised, as an outcome. It is not always recognised or made explicit as significant. But its production is evident across all the spaces in which medical research takes place. In our ethnographic field-site, for example, we witnessed the ‘radiation’ effect of scientific work in a form of self-knowledge referred to as ‘positive living’, vital for HIV+ workers' success as peer-educators, or in the intercultural communication skills national staff developed to accommodate transnational collaboration, and their adaptation to specific workplace styles – technical, linguistic and social. Epistemological ‘spillage’ radiated out even further, beyond the clinic into the everyday lives of research staff and participants, where we saw research participants draw upon trial benefits (such as transport reimbursements, trial participation certificates and new personal contacts in local health systems) to negotiate better lives for themselves. We witnessed it in the struggles of potential participants to navigate the landscape of medical research and intervention to secure the best possible healthcare in a fragile, uncertain system. We also saw it in the biographies of staff; in the choices they made regarding where they lived, how they lived, what they studied and what they aspired to. All of this, we argue, constitutes vital ‘scientific knowledge’.

Rather than excluding this type of knowledge from what is properly ‘scientific’, we consider it here in a continuum with more obviously ‘scientific’ knowledge of facts, technologies and apparatus. We explore the contribution that doing medical research makes to the broader lives of those involved, what scientific knowledge does for them, and what they can do with it in their lives in an African city. This means attending to the circulation of scientific knowledge beyond the confines of expert spaces and scientific production.

Common sense understandings of such extension of knowledge, as a transfer or transmission, diffusion or dissemination of knowledge, imply a movement of knowledge between places and people – global to local, experts to laypeople, science to everyday. In such renderings, knowledge remains (ideally) the same, while its ‘context’ shifts. If the knowledge does change along the way, this reflects simple distortion due to insufficient communication (e.g. Fitzgerald *et al.* 2002) or external factors, as in the case of misconceptions and rumours.

More sensitive studies of epistemological extension look for creative shifts and unexpected effects of mobile knowledge, questioning the idea of continuity in extension (Fairhead *et al.* 2005; Stadler *et al.* 2007; Simpson & Sariola 2012). We too explore the somewhat less expected knowledge that arises from trial participation. Our interest is directed at social and political-economic processes, and on the value of knowledge in the moral economy of HIV intervention (see Prince (2012) for a discussion of HIV and political economy). The question about extension is then less about the *transfer* or *translation* of knowledge from one group to another than about the *conversion of* knowledge from the operation procedures of a clinical trial and the scientific protocol into other forms of value, beyond that of universal scientific truth. Hereby, the problem of knowledge cannot be separated from that of social differentiation and economic practices – focusing on the clinical trial not as a matter of cognition but a site of value production (Cooper 2008; Kelly & Geissler 2011) and on knowledge not as a property but an asset continuous with, and convertible into, material conditions of life.

### Ethnography of an experimental community

The observations presented below were gathered during ethnographic fieldwork (2007–10) at the NCRO/CHA field-station, one of the largest and most prolific sites of bio-scientific production in sub-Saharan Africa. Located in a city with a population of *c*. 400,000, it is the largest private employer in the region with about 1200 staff. Running multiple clinical trials, epidemiological and observation studies and a health and demographic surveillance system, the station touches upon the lives of several hundred thousand people.^3^

### The city

With at least four other major transnational medical research organisations in addition to NCRO/CHA, as well as numerous smaller operations and university research departments, medical research activity permeates this city.

Transnational collaborative research began here after World War Two. NCRO/CHA collaboration pre-dates the HIV epidemic by 20 or so years – malaria is another of the region's disease burdens – but has rapidly intensified and professionalised since. This also reflects a wider HIV economy. By 2011 there were 117 NGOs with headquarters in the city. Fifty per cent reported HIV as their main activity.^4^ Previously a calm but important trading post, the city entered a period of decline in the 1980s/1990s with reduction of growth in key industries like fish/cotton processing and breweries. Today economic recovery has taken the form of an economy fuelled by HIV, as well as a major oil depot and new airport. Investment is evident in new hotels and shopping malls, but precariousness is still a feature of everyday life. The city's uneven history is reflected in its physical landscape and city spaces (Geissler 2013).

### Methodology

Our anthropological study was referred to as the ‘trial community study’, reflecting the idea that all those connected by science work – academics, funding agencies, policymakers, government doctors, researchers, participants, their relatives and the wider public – constitute a ‘community’, albeit a shifting, heterogeneous community bound together by multiple over-lapping networks across levels of scale (Geissler 2011). Our ethnography of this ‘community’ involved observation, conversations, individual and group interviews. We attended clinical and laboratory procedures, staff meetings, training and conference travel, as well as informal visits and social activities with staff and participants.

Our team consisted of ethnographers and assistants from Africa and the Global North, involved at different times. We lived in various city suburbs, including gated communities popular with expatriates and estates popular with science workers and participants. From these spaces we observed not just the specificity of NCRO/CHA in which we were embedded but also the wider landscape of HIV research and intervention in the city.

We interviewed over 100 staff involved in NCRO/CHA HIV research projects and conducted extensive, repeated, interviews with 89 HIV+ women participating in a study to prevent mother-to-child HIV transmission. The trial involved over 500 women recruited from antenatal care centres upon HIV diagnosis and followed for two years after delivery. We began interviewing these participants towards the close of the study, when some were still attending the clinic, and others had already ‘exited’. We followed several participants after the end of their participation, visiting them at their (often shifting) homes, and joining them during their visits to public HIV treatment centres.

## EXPOSURE AND EXPERIMENT

### Developing oneself

Being here [NCRO/CHA] I consider a step forward. It's a kind of an exposure; I am able to develop myself. (Male senior staff member)

Our interest in the movements of scientific knowledge, linking people's daily life in the city, personal and professional biographies and scientific work, found an apt expression in a term oft-cited by our informants: ‘exposure’. Many informants described how participation in medical research provided them with exposure to new knowledge, including medical, pharmaceutical and technical expertise, as well as exposure to organisations and places, people and lifestyles, processes and connections.

For staff, this included opportunities for formal higher education, but also access to less formal knowledge: exposure to new ways of thinking, technologies, practices and networks, and imaginaries of futures engendered by this exposure. A third of staff directly cited ‘exposure’ as a key benefit of working in this leading transnational research organisation. Others used different words to portray their exposure to formal and informal knowledge through their employment. They spoke of meeting new kinds of people and of learning how to interact with them (e.g. foreign scientists). They referenced previously unseen connections and procedures (e.g. grant mechanisms), hitherto inaccessible places (labs, hotels, international conferences) and objects and resources previously out of reach – such as computing, internet or state-of-the-art laboratory equipment.

‘Exposure’ thus offers itself as a broader cover for this much coveted mode of partaking in scientific knowledge. Working with NCRO/CHA – the leading research organisation in the region, as well as a key site of HIV policymaking and intervention – afforded staff the ideal setting to get exposed, partly because of the cutting-edge knowledge that research work produced, and partly because of the extraordinary power, resources and expertise invested in this collaboration. Exposure, as a rather nebulous form of knowledge-making, is particularly sought after and indeed vital in the contemporary post-HIV economy. In this new economy, formal professional training and higher education are somewhat devalued, and rapidly shifting knowledge-clusters, shaped by particular institutions, funding trends and fashions (e.g. HIV counselling and testing, ‘family life training’, male circumcision, peer education) are vital for finding employment and sustenance.

However, being ‘exposed’ also echoes older local meanings, sharing connotations with a term common since colonial occupation in many parts of Africa: ‘enlightened’ (Mayer 1963; Meinert 2009). Linking schooling with church and the production of modern upstanding citizens, ‘enlightened’ refers to being versant in modern technologies, able to handle wider connections, and, in consequence, better equipped for modern society and labour markets, as well as morally superior. One could say that ‘exposed’ has to some extent replaced the use of ‘enlightened’ between the educated post-independence generation (now retired to the countryside) and contemporary urban youths such as the NCRO/CHA staff. The encompassing connotations of exposure, like its predecessor term, referring to the whole person's betterment, is captured in the analogy of developing a photograph, sometimes made explicit: ‘it's like how you expose a chemical to the sun!’ Or in the words of a female laboratory technician: ‘I feel like I am almost fully developed.’

Exposure was not confined to staff. Research participants also sometimes deployed this term to describe knowledge – particularly around HIV self-care – gained through trial participation. But becoming exposed is somewhat different to the notion of being ‘empowered’ prevalent in HIV policy narratives about self-care (Prince 2013; UNAIDS and the African Union Joint Report 2015), which has a narrower focus on inner selves and individual rights. Exposure has a wider reach and, importantly, is a term with a chancier and more unstable quality. This is both its value and its risk.

The value attributed to exposure in the narratives of research staff and participants, therefore, cannot be divorced from concrete material concerns with making-a-living in a city with one of the highest HIV prevalences in Africa, a widening gap between the few in employment and the many outside and an ever-present sense of precariousness. When we look at how researchers and participants, such as the two described in the opening of this paper, make use of scientific knowledge we are also asking about what it is that makes a life. How do the people whose lives we have caught a glimpse of in our fieldwork imagine a meaningful life for themselves, their dependants and fellow citizens? What is it that drives them forward against the odds?

### Another kind of ‘exposure’

Securing a relatively stable existence in this city is challenging for many. So far we have referenced ‘exposure’ in relation to opportunity, but there is another kind of exposure faced by people: exposure to uncertainty and danger. Poverty, hunger, accidents, violence, illness and particularly, in the parlance of HIV research and interventions, ‘exposure’ to HIV infection. HIV prevalence is double the national estimate. Many people depend for their daily survival upon antiretroviral treatment, which has been provided free since 2005 through public facilities but which still reaches only part of the HIV+ population, and has important limitations in terms of organisation, diagnostic facilities and drug availability. Risk of exposure to HIV is a felt presence for everyone, as well as, of course, the motivation and precondition for the high intensity of HIV research.

Daily efforts to live in spite of HIV epitomise a wider economy of uncertainty: an economic order in which life has to be sustained day-by-day and where it never is certain whether needs – medical treatment, food or housing, children's school fees – can be satisfied the next day. Regular jobs as industrial workers or government employees – once a safe option for every school leaver – have become an exception for younger people, while school leaving certificates have lost much of their former value. Most people work in the informal or ‘jua kali’ (lit.: exposed to the hardship of the ‘fierce sun’) sector without a regular employer or stable income, and with minimal capital turnover or overheads. ‘Jua kali’ work was originally associated with making and repairing cheap, useful products outside traditional factory settings, usually along the roadside, but has grown to include all those ‘hustling’ for a living by, for example, hawking clothes, selling small quantities of vegetables or otherwise creatively trying to find something to do when locked out of formal employment structures (see Lourenço-Lindell 2010).

In our city this kind of unstable work is not only the province of the very poor or uneducated but also comes in professional-looking guises – at times celebrated as the new African entrepreneurial middle-class – that belie profound instability: itinerant computer repairers, insurance agents, self-employed pharmaceutical representatives etc.

Most of the female trial participants we interviewed lived such precarious lives. They were recruited from government antenatal care facilities, suggesting they had low incomes and no private medical insurance. Of the 89 women we interviewed, two thirds (67%) supported themselves, or were supported by husbands, through ‘jua kali’ work, such as tailoring, ‘mobile’ hair plaiting, or selling vegetables in minute quantities. Husbands' work involved unskilled construction or touting for buses. All these ‘small small business’ activities tended to be short-lived and shifting.

Of the few trial participants not involved in such work, three were supported by relatives and six were living off their domestic farms. Five had husbands with more regular employment: as drivers, a shop assistant and a printer's assistant. None of these involved regular payments. There were two nursery school teachers and one school secretary, all in low-status private schools, receiving inconsistent cash-in-hand payments. One mother was enrolled in teacher-training, and one a government primary school teacher. This mother and another who was a government driver were the only mothers whose families had any contractual link to a formal organisational structure and accessed a regular salary paid into a bank account.

Many mothers also experienced social insecurity. Few could describe their marital situations as stable and not infrequently marital arrangement changed during trial participation: there were many widows, second or third wives, women describing themselves as separated from their husbands, single mothers and wives out of formal wedlock, who could not count on husbands. Understandably, these mothers valued what study participation could offer them in terms of free, good quality healthcare, occasional material benefits such as water pots, transport reimbursement and generally being taken care of.

These small-value transfers are part of a range of new opportunities available to poor, especially HIV+, people within the local HIV economy. Boundaries between research and intervention are porous, since many scientific and academic institutions have expanded from clinical trials into implementation and evaluation. NGOs and ‘Community Based Organisations’ have become a dominant entrepreneurial model providing diverse economic opportunities. Some of these involve short-term contracts creating new specialisations in novel forms of expertise like community mobilisation and representation, quality assurance, participant retention etc., which in turn have generated high demand for training and certificates from new educational institutions providing certification in ‘HIV Counselling and Testing’ or simply ‘Community Work’. While employment in the non-governmental HIV sector is limited to those with formal education, high motivation and some good luck – and connections and access to information (‘exposure’) – many other people try to benefit from the HIV funds that circulate through today's urban economy through even less stable, ‘jua kali’-style activities, including providing services, housing and transport, as well as various forms of ‘volunteering’ such research participation (for more on the wider African volunteer economy see Prince & Brown 2016), and the formation of self-help groups for the sake of ‘income generation’. In 2011 there were 10,172 registered Women's groups, 16,353 Self-help groups and 1,105 Youth groups in the city^5^ – an unlikely one group per 15 residents.

Perhaps more important to this economy than immediate material gains are the potential, imagined, possibilities of finding an inroad into the success associated with NGOs and large research organisations– big cars, smart dress, private healthcare, sponsored education and travel. When we asked younger people in the city without links to NCRO/CHA whether they had ever applied for work with NCRO/CHA and most answered ‘yes’ accompanied by resigned laughter. Many applied numerous times, sometimes for jobs beyond their educational credits.

### Working class

Those who do manage to enter the sector formally become members of what is known as the ‘working classes’ (those working for a regular salary). The staff we interviewed are leading representatives of this class. They are all secondary school graduates and include medical doctors, nurses and laboratory technologists, and others with more specific skills such as trial mobilisation, or HIV testing and counselling. Competition for positions is high – with 50–100 applicants even for menial jobs. Previous experience in research or NGOs is an essential asset, documented in candidates' thick portfolios of certificates. In comparison to many city residents, staff are already ‘exposed’. Their employment provides them with a degree of security, including written contracts, salaries paid into bank accounts, and health insurance.

However, in some respects they are in a very different position to the original, post-independence ‘working classes’ of teachers and civil servants; their parents' generation (the ‘Enlightened’). While these had spent most of their lives working on permanent pensionable contracts with government ministries, NCRO/CHA staff have at best one-year contracts, with higher perks but without lasting tenure and secure retirement, and their salaries and promotion depends upon foreign institutions with shifting expatriate staff and changing policies and budgets.

Fundamental uncertainty therefore applies thus to everyone in the city. In some respects, the line between an HIV researcher and a woman surviving on ARVs and selling tomatoes by the piece is porous; both lives are contingent on the vagaries of international donors. Even those who, from a poor participant's perspective, have ‘made it’ into employment remain ‘exposed’ to uncertainty. Transnational medical research exists in a state of permanent temporariness – or ongoing boom and bust – dictated by the politics of international funding, project cycles and of course the science and urgency of the disease burden itself. Staff, thus, expressed considerable disquiet around the time of the annual renewal of one-year contracts and the completion of research studies. If a contract ends or a new study does not immediately follow a completed one, private medical insurance is stopped, cars are sold and children's school fees are at risk. To enhance stability and diversify options staff, like participants, run (as noted in the opening vignette) businesses on the side. These range from the substantial or skilled, investing in shops, farmland and rental properties, to the same ‘small small’ businesses as their participants, i.e. hawking clothes, vegetables, soda and mobile phone scratch-cards to colleagues.

### ‘Just trying’

What most current economic activities in this city have in common, then – including even HIV research employment – is short-term temporality, dependency upon outside funding sources and resulting uncertainty. As a common expression in the city puts it, one is ‘just trying’. While direct, literal struggle for survival certainly is not the condition of life for everybody, this extreme – and the spectre of death as the ultimate certainty underlying it – affects almost everybody's life. Everybody has relatives living under such conditions, and fulfils attendant responsibilities, and in the absence of social security, many are at risk of sinking to the level of mere survival. Life is a constant process of trying, of pursuing possibilities without knowing their outcomes (see Whyte 1997); an ‘experimental’ life, exploring uncertain, innovative ways forward, that at least as often fail as succeed.

In this African city, then, ‘exposure’ takes two very different meanings – danger and promise – as does ‘experimentation’, which can refer to the externally controlled clinical trials that provide the frame of this ethnography, and to daily attempts by inhabitants, including those collaborating on clinical trials, to move forward, ascertain sustenance and create some security in an uncertain terrain. It is against this background of exposed lives and everyday experiment that we now explore specific ways in which both research staff and participants ‘convert’ scientific knowledge into strategies for moving forward in life.

## SEEKING EXPOSURE: STAFF

### Pursuing degrees

Obtaining further formal education was an important part of seeking exposure for staff. NCRO/CHA is a scientific institution, where workplace hierarchy reflects academic degrees. Certificates, diplomas and Masters degrees featured heavily in future plans and as reasons for joining NCRO/CHA, which had an educational sponsorship programme.

Often future plans were expressed in terms of reaching somewhere distant, e.g.: ‘my aim is to be a Principal Investigator! Ha ha!’ (female community fieldworker) or ‘I got really inspired and said “I want to be an international policymaker!”’ (male nurse). Although such statements tended to be accompanied by ironic laughter there was an idea, supported by widely narrated rags-to-PhD biographies within the organisation, that such distant places could be reached through careful step-by-step planning and effort.

The field station offered some limited funding towards diplomas, Masters and even PhD studies. However, relatively stable wages and a yearly gratuity, made self-funding an option and many staff were enrolled in privately paid academic evening courses. NCRO/CHA also provided invaluable infrastructure for distance learning: high speed internet, new computers, professional mail addresses, subscriptions to literature databases that few African university students can access, and research data for thesis work. To utilise these resources for their ‘schoolwork’ and other assignments, staff often stayed late at their offices. Beyond material support, this international, high-aiming workplace also provided crucial exposure to knowledge about courses, their content, reputation and employability potential in a rapidly shifting market of HIV expertise, application procedures, deadlines, styles and references. NCRO/CHA thus provides a good *placement*, a position that opens up future opportunity, and provides an overview of networks and the terrain of possibility, as well as contact with others who could provide strategic and academic advice. This sort of learning is not simply a matter of ‘*who* you know’, but knowing *where* to look, and when, and also of how to make oneself known, visible to opportunity, potential supporters and gatekeepers.

### Learning on the job

Some directly research-relevant knowledge, such as computer skills, lab techniques, technical terminology or clinical handling procedures, was conveyed as part of institutional ‘capacity building’ by senior staff or external experts or, in the case of laboratory equipment, by the manufacturers. Other skills, notably in scientific dissemination, were acquired through paper-development workshops or conference presentation rehearsals.

Most staff's scientific knowledge, however, was gathered informally, through conversations, or observing others. Here, the concept of ‘exposure’ with its connotations of learning through being somewhere is particularly apt. Large organisations, notably transnational ones, develop their own sociality and work-related cultures; and working for such organisations impacts people's lives through formal and informal learning processes and habitual inculcation to work ethics (Wright 1994). In NCRO/CHA this involved knowing how to be a good researcher – in the sense of proper scientific conduct, and adequate discursive frames, such as the bioethics rhetoric of ‘autonomy’ or ‘confidentiality’, or the idiom of ‘going the extra mile’ to achieve valid scientific results and satisfy senior staff. Staff learned routines such as daily work rhythms and meeting procedures, and, not least, appropriate styles and manners of self-presentation. They described for example how they, in their interactions with expatriate management, had learnt to reference authority without appearing deferential, make their voice heard in professional meetings, and display personal initiative and independent thinking, which they found more highly valued, or expressed in different ways, than in their earlier experiences, e.g. in government institutions. Skills in intercultural communication and adaptation to the idiosyncrasies of a Global North government institution also extended from the workplace into the private domain – involving exposure to party behaviour, leisure and sports activities, or gift giving. Significantly, staff often helped each other, sharing and discussing ideas, reading each other's university coursework as well as offering support during family tragedies and – for those with clinical skills – sometimes providing informal expert advice and treatment to colleagues and their relatives.

‘Being with’ NCRO/CHA therefore involved habits and connections, ranging from preferred social venues and shopping places, gained and lost friends and social networking, to the embodied dimensions of speech, posture, hairstyle and dress. Some of these aspects remained implicit, others were consciously experienced, and either valued as exposure, or registered with some irritation; thus, some staff recalled how, during the initial days of their employment, their dressing styles and comportment had made them feel out of place among the well-groomed staff: ‘I could hear my flip-flops in the staircase, among all these high heels’ (female nurse). The same staff member likened the totality of the experience of entering the new work culture as: ‘You feel like you are in another world, you are in another country.’

Pushing this analogy further, informal learning within this workplace also involved getting to know new people at the workplace and beyond – globally and locally: ‘I have exposure. I have met so many people that I never knew that I could meet in the world of HIV/AIDS. I have talked to them. I have shared with them. I have learned a lot' (male community technologist).

The pleasure that this staff member – who otherwise was particularly critical of the inequalities and shortcomings of transnational collaboration, even evoking its ‘colonial’ continuities – took in making connections across continents, national boundaries, race and class, was reflected by his screensaver displays, depicting him in various exotic locations, sharing moments of conviviality and touristic experiences with world-leading HIV scientists at international conferences.

### ‘Fitting into the world map’

Thus exposure to scientific knowledge and attendant modes of being – in conferences, seminars, planning meetings and discussions with senior staff and overseas experts – implies a process of becoming part of a larger world: ‘We have access to literature materials about youth intervention, about research work that is being done in many places, we are in a sense being fitted in the world map of adolescent sexual reproductive health' (male doctor).

Sometimes exposure to the wider world was through literal *mobility* as in the case of staff given the chance to travel to international conferences. Such opportunities were highly valued, not just for the chance to mingle with experts but also to see and experience more of the world and exchange ideas with others in similar situations. Under wider conditions of globalisation, mobility itself is often associated with the realisation of value. Some of the value realised or, we could say *converted* through scientific knowledge, was less lofty than the exchange of scientific ideas. One staff member for example, was initiated to the global clothes trade when talking with colleagues at a conference in Thailand, enabling her subsequently to set up a successful clothes shop in the city. A few staff even ‘got lost’ overseas, using temporary visas obtained through international conference participation to start new, potentially just as uncertain, lives abroad.

For the many who remained within Africa, these wider spaces and collectives, beyond the immediately accessible, were demarcated by senior staff's teleconferencing and international travel, data-faxing to research network headquarters, equipment supplies and maintenance by multinational firms, international technical standards and guidelines and reliance upon foreign grants and funders. It was present, for example, in the common reference to the CHA's Northern headquarters, as in ‘we are waiting for [HQ's] response’, and related turns of phrase like ‘back there’, ‘out here’, ‘coming down/out’, with implications of a global, hierarchically structured space, or in the disembodied voice of a foreign ‘principal investigator’ emanating from a mobile phone at the centre of the meeting table. The imaginary of other (better, higher) places combined with the value placed on further education, noted above, into a specific vision of moving forward in life, out of one's present condition towards new places.

CHA has a particular place within African HIV and bioscience landscapes – conducting not only large-scale, world leading research, but also shaping health policy across the continent, and implementing HIV care and treatment through government institutions. Attachment to NCRO/CHA provides an opportunity to work and learn in an environment that represents, like no other in Africa, the cutting edge of scientific – as well as clinical – expertise and possibility. It is the best place to get reliable information on the latest global scientific developments, and develop new ways of thinking about the world – a site from which both the world and one's place in it, and oneself, are transformed.

The recognition of the state of the art, inherently influential positioning of NCRO/CHA's scientific knowledge was not simply a matter of personal career advancement, good salaries or per diems. It also speaks of a search for innovation, potency, trustworthy knowledge and global standards, and reflects staff's curiosity and yearning for job satisfaction: being able to do what one knows one could and should do (see also Wendland 2012; Aellah *et al*. 2016). This was particularly the case for clinicians who, equipped with drugs, diagnostic equipment, laboratory facilities, and sufficient time for clinical encounters, felt able to perform their clinical commitments to their full potential and generate and engage clinical knowledge:

I've learnt about good clinical practice, about how to do certain things in research… Research is very strict in the ways that they do things. So, actually it builds discipline in you, I think I have loved that. (Female clinical officer)

Comparing research work to his previous government work, another clinical officer described how he now had the chance ‘to think about patients differently’, having the time and resources to get to the bottom of difficult cases, including the time to talk to patients' spouses and understand their social situations. Although these possibilities were partly due to the need for valid clinical trial data, the situation also afforded this clinician a valued chance ‘to treat clients so nicely’.

Satisfaction with doing good work – and doing good – also applied to other professions among the staff. A laboratory technologist described with pride how joining a research project exposed him to the newest technologies and techniques – ‘we always know the latest things’. As well as pride in being on the cutting edge, he spoke of research as instilling in him a desire to want to do things with scientific rigour, contrasting this to working in the government and private health sector.

On this note, the exposure to new ‘worlds’ through work as discussed earlier also included exposure to different realities on a local scale and changed sensitivities. For some staff who had grown up in more privileged circumstances, exposure to other local communities, social classes and social experiences was as enlightening as exposure to leading HIV scientists and experts. This was more pertinent for community-based staff working outside the lab or office: ‘I have got a lot of experience from this study, it has actually made me know that human beings have different needs and everybody is unique. I have come to socialise with the community. I have known a lot of places within the region.’

### The promise and challenges of graduation and conversion

Staff drew analogies with graduation when describing their time at NCRO/CHA: ‘This place is just a High School; we are moving on … Once we get through the High School we leave CHA and go to a different organisation in a different capacity, we go very far out there; so this is a learning place, a school until you get there’ (female supervisor).

The metaphor of ‘graduation’ – a particularly sought after social occasion in this part of Africa, immortalised by photographs displayed in every living room – underlines the forward-moving nature of knowledge, and it implies a vision where NCRO/CHA is a step on the way rather than a final destination. Employment with research is time-limited and will end, the positive outcome of which is to move ahead to a more permanent or at least even better paid job. The particular attraction of this knowledge, then, lies in its convertibility and transportability. It is of value in the city's HIV economy, which involves related organisational structures and similar procedures and idioms, such as notions of participant autonomy, voluntarism, standard operating practices, evaluation, participant flow, consent etc. which have travelled within the organisational networks of the HIV economy.

However, like the future after graduation, conversion is not always straightforward. The success of such movement depends upon the market situation, and upon the nature of the knowledge. In spite of the booming HIV economy there are increasingly many knowledgeable workers and competition among them is getting harder. When this is coupled with the ongoing boom and bust nature of project-based HIV research work, there were times when the harder, more painful, edges of the search for a better life revealed themselves in competition for roles in new projects. When some staff lost out mutters of patronage, sexual favours and corruption surfaced and the ethos of conviviality was harder to maintain.

NCRO/CHA's staff's pursuit of scientific knowledge in their city's leading institution for knowledge making and dispersal, and their emphasis on the value and marketability of such knowledge may, therefore, seem individualistic and self-serving. This recalls other ethnographic descriptions of African donor-funded AIDS programmes, which according to their Northern authors are inhabited by ‘aspiring’ elites involved in ‘hunting and gathering in a terrain of AIDS NGO projects’ (Swidler & Watkins 2009: 2), in order to ‘remain buoyantly suspended above the froth of projects and donor interests through good contacts and placements’ (Swidler & Watkins 2009: 10). However, the pleasure taken in good work and skill development, described by staff above somewhat runs against a depiction of utilitarian ‘hunter-gatherers’. Moreover, staff's social conscience and civic commitment are also expressed through doubts held by the staff concerning connections between research and society, and between global standards and technologies, and local possibilities (see also Feiermann 2011). Some staff – especially in the laboratory – expressed doubt of ever returning to government work and circulating their newly gained knowledge in the public health sector, because of lacking resources to utilise their knowledge: ‘Government is frustrating for many, many reasons; you don't have things to work with … and if you are someone who is lazy and you just want to pass time … you can get away with it’ (male laboratory technician). But, importantly, this was often phrased as an uncomfortable thought. One supervisor, for example, emphasised that the technological and pharmaceutical possibilities within NCRO/CHA work were such that one was at risk of ‘forgetting’ about Africa:

But sometimes I say CHA can cheat you until you forget that out there in the world, everything is not like CHA. It makes you feel so comfortable in your work, that it might even hamper your creativity; you might start thinking like a non-African and look at the world like a non-African. But my own perspective is, that if you are in Africa and you are dealing with Africans you have to think like an African.

This quote illustrates a characteristic yearning for civic responsibility. Indeed, some staff, notably clinicians, developed, though their work experiences, a critical outlook towards social injustices and their health system, shaped by exposure to institutional inequalities and by facing the discrepancies between medical possibilities in transnational research and their inaccessibility to most Africans (see Wendland 2012).

### Exposing others, opening futures

Another form of further extension and conversion of scientific knowledge is visible in staff members' attempts to convey their own exposure to their children. Drawing upon their relatively stable financial resources and awareness of educational opportunity, many staff prioritised the education of their children in their future hopes: ‘I have so many dreams, [laughing] I want my boy to go to the best school’ (female administrator).

In addition, most staff have taken on responsibilities for orphaned children of their kin. Thus, one nurse described how she is ‘pulling’ family members ‘up behind her’ as she progresses within medical research. Her family was decimated by HIV leaving many dependants in her rural home. Her aunt sponsored her secondary education and certificate in nursing, hoping she might become a future stabilising influence in the family. After experiences in mission hospitals she was employed by NCRO/CHA and after initially self-funding her further education, she eventually received sponsorship for Masters studies. Using her salary, the annual gratuity, and per diems saved from conferences, each year she gradually built up the number of relatives whose education she sponsors. Underlining the value of education, she compared herself to her brother who stayed home, married young, and now has several children. Yet she convinced even him to go back to finish school and start this process for himself and his own dependants. Other staff, too, talked about this social conversion of knowledge with pride: ‘I can be proud because I am now furthering my education, and then I am supporting some other people, and I also intend or plan to support a pupil next year, who is joining secondary. So of course I account those as achievements' (male records-keeper).

Often, like in the above case, this process of conveying knowledge starts as soon as one earns a salary. This is a tough and uncertain effort. It involves careful budgeting, saving and triage, as well as facing unreasonable expectations and the possibility of educational failure. Working at NCRO/CHA is particularly useful, not just because of the higher wages, but because staff know what knowledge counts where and where best to attain it.

## SEEKING EXPOSURE: RESEARCH PARTICIPANTS

### Peer-educators

As discussed earlier, the increase in HIV funding and the associated growth in income opportunities has created a generalised imaginary of possibilities among all city residents. The HIV+ mothers we interviewed represent – in the most part – a distinctly different economic and social class to the staff discussed above. Possibilities open to them for exposure and career development are, therefore, markedly reduced. However, there was one area where study participation, in conjunction with one's biological status as HIV+, was an entry point to a form of employment, albeit often as inconsistently paid ‘volunteers’. This was the field of ‘peer-education’.

Peer-education is the use of HIV+ ‘peers’ for counselling, care and general assistance in both research and treatment. Peer-education with HIV treatment centres represents a particular kind of ‘voluntary’ labour in this city. While officially ‘volunteer’ positions, most of the time volunteers receive a regular monthly payment and sign contracts. More significantly, volunteers are exposed to the formal workings of the organisation. They have to adhere to specific working hours, attend staff meetings and, to centre clients, appear as regular staff. In their day-to-day encounters with clients, they are holders of expert knowledge on HIV. Occasionally chances present themselves for some training or additional paid work, such as mobilising during mobile VCT sessions. Some peer-educators can make the transition to a formal contract as community health worker with the treatment centre, although this does require a school leaver's certificate.

Seven of the 91 mothers we interviewed became peer-educators in HIV research or treatment programmes after they exited the study and before the end of our fieldwork. Three were employed on temporary contracts by the study itself to act as peer-educators to other participants. They were later absorbed as regular staff on other studies or moved on to better paid positions in external organisations. Others were working in various HIV treatment centres in the city and beyond. Peer-education as an economic opportunity for this group takes on significance in terms of both numbers and imagination when it is noted that this was, aside from one teacher and one government employee, the *only* actual employment held by any ex-study participants we interviewed.

Study peer-educators were generally valued for providing tender care, and spoken of lovingly by other participants: ‘they were encouraging our hearts’. They also held a somewhat iconic status for both staff and participants – as a representation of what could be achieved and the potency of exposure. When one ex-study peer-educator who had obtained a car was spotted driving, both another participant and a doctor remarked on how well she was doing: ‘She is even driving a car now!’

This particular peer-educator had used her salary to gain various counselling certificates and eventually applied for jobs in other organisations. In the time that we knew her she moved onwards and upwards several times, dropping ‘peer’ from her job title and developing a career trajectory indistinguishable, from that point on, from other staff. By 2016, when we shared a draft of this paper with her, she told us she was now a degree holder and social science interviewer herself with 8 years of experience working with various transnational research collaborations, commenting ‘It's amazing. I really never thought that I was a source of encouragement to many. I mean, it is just totally amazing.’ This peer-educator thus represents an imaginable but idealised and infrequent endpoint that creates a continuum between staff and participants in the circulation and conversion of scientific knowledge.

### Placement

The study peer-educator described above seized upon her physical placement within the research organisation as an opportunity to capitalise on her exposure to people she might normally have not met:

I used to say to myself: ‘God, when my boy reaches six months I don't want to be here and I want the study to give me a job so that I can be able to give him formula.’ So that time I shared it with the (trial) doctor. She encouraged me to go and see the Principal Investigator. So I carried my baby, I carried my umbrella and I was so tiny. When he was called, he came in the reception. It was so nice. He carried my umbrella and my bag. Then I explained to him what I wanted. He looked at my CV. There was a project I had done for a CBO and it really excited him.

This action – and subsequent changing trajectory of placements – was a source of inspiration that also, ironically, created some distance between her and some participants who couldn't make the same use of their placement in the trial:

We used to share the [waiting room] bench … but it sometimes happens to many people that when somebody is employed she changes. I found out we can't rhyme together [anymore] so I just kept away. She was picked to be a peer-educator because she was suffering. I felt bad because everyone is in need. Should I also go and say my problem so I can be employed? But my legs were just heavy for me to go.

The importance of placement as a way of opening up further opportunity was also experienced by participants who became peer-educators in HIV treatment centres after the study ended. Like staff, by virtue of being in a space of activity in the field of HIV, they had occasional chances to gather useful information. Sometimes this worked in rather refracted ways. The study had created a self-help support group for participants and encouraged them to join both for emotional support and as an opportunity for talks and small training sessions (safe water, memory books etc.). This inspired one mother to join a similar support group attached to her HIV treatment centre after exiting the study. There she became friends with one of the centre's existing peer-educators. When an advert was placed on the wall in the centre for new peer-educators, it was he who told her when and where to look.

### Acquiescence of nomenclature and skills

Study participants received no formal training in HIV knowledge. But informally, in the process of being a study subject, undergoing clinical procedures and receiving counselling, learning the rules of the study and sharing with other participants and study peer-educators, they did gain specific knowledge that was not only of lasting use as an HIV positive person, but also convertible into social advancement for those that became peer-educators. One of these participants described how she had been asked to explain the terms ‘adherence’ and ‘opportunistic infections’ when she interviewed for a volunteer job, terms she knew very well because the study had been strict on adherence and had encouraged participants to come to the clinic frequently to treat opportunistic infections. A treatment centre coordinator, in turn, told us that she had specifically asked another participant to become a peer-educator because she saw from her referral letter that she had taken part in a study and would therefore be ‘knowledgeable and disciplined’. The use of the term ‘discipline’ is important here. As role models for others peer-educators are expected to be experts in their own self-care.

### Certification

The convertibility of implicit study knowledge was made explicit when participants asked their study doctors to confirm their knowledge in writing. When one mother who later became a peer-educator at her treatment centre responded to an advertisement for community health workers shortly after the end of her study participation, she went, like several of her fellow hopeful ex-participants, to visit her study clinician because: ‘I wanted the doctor to write for me at least a certificate to say I am from the study and know something about HIV, because they wanted a school certificate and I don't have one.’

Similarly, one of the ordinary study participants we visited proudly showed us her file of certificates, including a copy of her study referral letter (issued at the end of the trial to refer her for post-study HIV treatment) and her study ID card, side-by-side with her school records, letter of reference for a job as a petrol station attendant and the certificates for workshops on ‘HIV Home based care and nutrition’, ‘Memory book making’ and ‘Taking care of the elderly’ that she had participated in as a volunteer community health worker with a church-based NGO. This folder was a visible representation of her own valuation of the place and convertibility of scientific knowledge gained through study participation in her life.

## ACCUMULATED EXPOSURE

Research participants' opportunities to convert knowledge are small and unsteady, compared with those of staff. But they can accumulate, as in the following case of a participant-turned-peer-educator:

In school Maureen had dreamt of training as a nurse. But when her parents died she was left to look after her sister, forcing her to leave school. Rejected by relatives, she struggled to find somewhere to live. After telling a shopkeeper her life story, she got a job but was often sick. She informally married a ‘jua kali’ man who paid her hospital costs. When she became pregnant she found herself to be HIV+ during her first antenatal visit, where a NCRO/CHA staff recruited her for the trial. When she joined the study she knew nothing about HIV. Described as a ‘good participant’ by staff, after exiting the study she went to continue treatment at a donor-funded centre, where she joined a support group and befriended a peer-educator, who alerted her when they advertised for peer-educators. At interview she described her experiences in the research study and, as she proudly recalled, was able to answer confidently questions such as ‘Do you know the work of Septrin?’ She got the position and was paid the equivalent of one day's informal domestic cleaning work per day. Later, international funding was stopped but she continued working voluntarily because she thought this better than sitting in the house where she ‘might be forgotten’, invisible to other opportunities.

She continued to learn the work of the centre, being deployed in the pharmacy, the records department, and helping in drug adherence training for HIV clients. She also benefitted from improved quality of medical care, having direct access to doctors and her patient file to make sure her lab results were returned in a timely fashion.

She continuously hoped for employment: ‘So we thought maybe these people the way they are appreciating us they will just employ us.’ When she applied for a job tracing drug defaulters she went back to a NCRO/CHA study doctor to get a letter of recommendation based on her study participation. But she was eventually told by the treatment centre human resource manager that any employment would require a school leaver's certificate. She thus started secondary-school education at an evening school, partially sponsored by an expatriate researcher she had met during the study. Today she dreams of eventually getting her school certificate, becoming a community health worker or researcher and ensuring her dependants can go to school and beyond. This dream is a long way off. She is still working unpaid.

Maureen's story shows the diffuse and fractured nature of exposure and conversion of scientific knowledge, but also the potential of its cumulative effect – Maureen has moved on, at least a little, from where she started. It also shows its limitations and barriers, particularly related to socio-economic inequalities, captured in the following encounter between Maureen and a client at the treatment centre:

A smartly dressed client came to rearrange her next visit because she had a higher diploma exam in the capital city on that day. She encountered Maureen who, during the in-charge's absence, was registering clients and addressing queries. Carefully, and with quiet authority, Maureen told her this would not be possible. She explained the importance of the two-week visit after initiating ARVs, checking that drugs were working and monitoring side effects. She suggested the client should come early in the morning before travelling to the capital. Upset, the client tried to negotiate, but Maureen stood firm. Eventually the client left in a huff, saying that she might fly to the capital to get there in time for her expensive exams. In contrast Maureen, who walks to the clinic to save the bus fare, returned to work, her books for the first year of secondary school in a bag under the desk. ‘She probably won't come back’, she told us, ‘she doesn't understand the importance’.

### Beyond Peer-Education

Some of the processes of exposure and conversion described in relation to the peer-educators were also experienced by the participants who did not achieve such status. The most obvious is the exposure to knowledge about one's HIV status and self-care developed through study participation. One participant described this process – of despair when discovering her HIV status to learning that there was treatment, and that she could have an HIV-negative baby – within a terminology of moving from darkness to light: ‘By then I didn't know anything. I only knew that once you fall positive yours is only death. I was still in darkness. But they explained to me the benefit of taking the drugs and the study, now I just thought I could help the life of my unborn.’

Much of this knowledge was circulated not through direct teaching or dissemination but through the social experiences mothers gained whilst waiting on the clinic benches. Here, mothers learned that there were others in their situation, and felt encouraged that they were not alone and could live a normal life.

Producing an HIV-negative baby at the end of this HIV transmission prevention study could be seen as the ultimate conversion of scientific knowledge that participants could gain through study participation. In dissemination meetings the scientific results of the study – the very significant reduction in HIV+ babies – was thus celebrated as a scientific triumph but also a personal one, achieved through hard work, steep learning and strict adherence to the new knowledge. It was notable that when we visited those mothers whose babies had become HIV+ during the study, we generally did not find, as we had expected, scenes of despair and tragedy, but rather scenes of quiet hope, organised effort and loved children. These ‘exposed’ HIV+ children were being looked after by ‘exposed’ mothers, armed with new knowledge about how to make a life for themselves in this uncertain city.

There was a myriad of other, small, ways that study mothers found to take something of value from study participation and use it beyond. A few mothers, for example, opted to join other studies when the chance arose, having seen the benefit; others used their experience of high quality care, as well as discussions with other mothers to make informed decisions about which post-study HIV treatment centre to attend, even occasionally changing centres or coming back to consult with study doctors over issues in their care. Here, the importance of making use of social connections in a healthcare system perceived as contingent on relationships and placement is highlighted (see also Reynolds Whyte & Etyang Siu 2014).

## FUTURES

What kinds of futures are imagined and imaginable by the different trial community members? Concerns about maintaining lifelong access to donor-dependent life-saving HIV drugs as well as meeting their families' basic needs in a precarious economy continue to infuse day to day life for trial participants. However, the value placed on exposure to scientific knowledge reflects ways of trying for, and dreaming of, more certain futures. Trial participants' carefully collated folders of trial participation certificates invite us to consider what it would mean to take seriously the yearning for knowledge among all members of the trial community – including those whose participation at present is of a purely somatic nature. Could one utilise the opportunity for scientific and medical extension work afforded by medical research participants' attachment to knowledge-producing institutions (for periods that are equal to, or even exceed, the duration of common higher education courses)? Could one imagine then that transnational research programmes – many of which now look back at decades of local clinical trial work, and consequently generations of ‘exposed’ populations – actually become formal sites of public engagement with and teaching of science?

Other trial community members have their own ideas about how to move forward and stabilise the effects of their exposure to scientific knowledge. During our time with NCRO/CHA a suggestion that the community advisory board, made up of representatives of city subcultures (motorbike taxi drivers, religious leaders, commercial sex workers, women's groups, government doctors etc.), should register itself as an independent body and act as a permanent filter between the city and any research project that wanted to work in it was repeated again and again in meetings. And in the concern of the supervisor who struggled to retain the capacity of ‘thinking like an African’, we see an ideal future dreamed as one where science in this city could/should be a centre positioned among centres, rather than a periphery or satellite of a centre in the Global north (see Moyi Okwaro and Geissler (2015) and Lachenal *et al*. (2016) for discussions of African scientists' perspectives on international collaboration in the context of asymmetric relationships).

## CONCLUSIONS

Our interviews and observations with African research workers and participants bring to view the value of ‘exposure’ to scientific knowledge in its broadest sense to those whose day-to-day labour and mutual collaboration are the foundation of transnational medical research. These effects are not limited to the long-term social good of scientific progress or to the immediate material benefits of trial participation, but extend to subject formation. Although we recognise that the structures of both global and local inequality and injustice within which medical research and healthcare operates exclude and stagnate as well as include and energise (see Sullivan 2012; Aellah *et al*. 2016), we argue that informal extension of knowledge from scientific work processes is explicitly valued and sought after by participants and staff alike, and we illustrate how such exposure, despite its chanciness, is convertible into better lives, ranging from gaining formal employment to much smaller movements in the daily search for a better life.

It needs to be stressed that these conversions of scientific knowledge between different productive settings in the lives of the staff and participants are vital *both* to the lives –sometimes the survival – of those working on the trial, and to the maintenance of the scientific production itself: without this process of extension, labouring for science – be it technical work or bodily participation – would be a lot less attractive to people living in the city. Without the benefits of exposure, the scientific production process would lose much of its privileged status among the employment opportunities in the city. Life science and everyday life are in this sense co-constituted, and the flows of scientific knowledge across the city link it all up and keep it alive. In African cities like this one, there is no clear separation of science and sociality. Transnational medical research has become a part of everyday social and economic life. In this city, it is no longer something new, imposed from outside, or somehow separate from ‘national’ everyday life. To staff and participants, it is, simply, part of life.

## NOTES

1. We used pseudonyms and representative composite stories. Direct quotes are from interviews but surrounding information has been changed. Verbal informed consent was obtained for participant observation. Written informed consent was obtained for interviews and focus groups. Our research protocol was approved according to the ethical review processes of both our then employers and all collaborating research institutions: NCRO, CHA, and a third overseas institute for tropical disease who was also partnering on HIV research and intervention work during our fieldwork.

2. The local ‘Centre for Global Health Research’ of the National Clinical Research Organisation (NCRO) was mostly financed by its collaboration with the overseas Central Health Agency (CHA). The NCRO Centre consisted mainly of the CHA ‘field station’ although it had several smaller collaborative projects in operation, independent of the CHA.

3. The total number of people contributing to, and living off, medical research is hard to determine. In addition to staff, research depends upon a corona of ‘volunteers’, working in return for casual payments and ‘reimbursements’ e.g. peer mobilisers, women's and youth groups involved in recruitment and education efforts, interns etc. The total number of people contributing work to the medical research endeavour and gaining knowledge and material benefits from it, would thus be several thousand, even before considering the trial participants’ somatic labour.

4. According to the local Non-Governmental Organisations Co-ordination Board.

5. According to the District Officer for Social Services.

## References

[ref1a] Aellah G., T. Chantler & P.W. Geissler 2016 Doing Global Health Research in an Unequal World: ethics case studies from Africa. Wallingford: CABI (open source). 29035486

[ref1] Cooper E. & D. Pratten, eds. 2014 Ethnographies of Uncertainty in Africa. London: Palgrave Macmillan.

[ref2] Cooper M. 2008 Life as Surplus: biotechnology and capitalism in the neoliberal era. Seattle, WA: University of Washington Press.

[ref3] Crane J.T. 2013 Scrambling for Africa: AIDS, expertise, and the rise of American global health science. Ithaca, NY: Cornell University Press.

[ref4] Fairhead J., M. Leach & M. Small. 2005 ‘Public engagement with science? Local understandings of a vaccine trial in The Gambia'’, Journal of Biosocial Science 38: 103–16. 16266443 10.1017/S0021932005000945

[ref5] Feiermann S. 2011 ‘When physicians meet: local medical knowledge and global public goods’, in P.W. Geissler & C.S. Molyneux, eds. Evidence, Ethos and Ethnography: the anthropology and history of medical research in Africa. Oxford: Berghahn, 171–98.

[ref6] Fitzgerald D.W., C. Marotte, R.I. Verdier, W.D. Johnson & J.W. Pape. 2002 ‘Comprehension during informed consent in a less-developed country’, The Lancet 360: 1301–2. 10.1016/S0140-6736(02)11338-9 12414207

[ref6a] Geissler P.W. 2011 ‘Studying trial communities: anthropological and historical inquiries into ethos, politics and economy of medical research in Africa’, in P.W. Geissler & C. Molyneux, eds. Evidence, Ethos and Experiment: the anthropology and history of medical research in Africa. Oxford: Berghahn, 1–28. 24524172

[ref7] Geissler P.W. 2013 ‘The archipelago of public health: comments on the landscape of medical research in twenty-first century Africa’, in R.J. Prince & R. Marsland, eds. Making and Unmaking Public Health in Africa: ethnographic and historical perspectives. Athens, OH: Ohio University Press, 231–256.

[ref8] Geissler P.W., ed. 2015 ‘Science in the African para-state’, in Para-States and Medical Science: making African global health. Durham, NC: Duke University Press, 1–46 (open access). 25834888

[ref10] Kelly A.H. & P.W. Geissler. 2011 The value of transnational medical research. Journal of Cultural Economy 4: 3–10. 21874135 10.1080/17530350.2011.535329 PMC3158132

[ref11a] Lachenal G., J. Owona Ntsama, D. Ze Bekolo, T. Kombang Ekodogo and J. Manton. 2016 Neglected actors in neglected tropical diseases research: historical perspectives on health workers and contemporary Buruli Ulcer Research in Ayos, Cameroon. PLoS Neglected Tropical Disease 10: e0004488. 10.1371/journal.pntd.0004488 PMC4839562 27101371

[ref11] Latour B. & S. Woolgar. 1979 Laboratory Life: the social construction of scientific facts. Princeton, NJ: Princeton University Press.

[ref12] Lourenço-Lindell I. 2010 Africa's Informal Workers: Collective Agency, Alliances and Transnational Organizing in Urban Africa. London: Zed Books.

[ref13] Mayer P. 1963 Townsmen or Tribesmen; Conservatism and the Process of Urbanization in a South African City. Cape Town: Oxford University Press.

[ref14] Meinert L. 2009 Hopes in Friction: schooling, health, and everyday life in Uganda. Charlotte, NC: Information Age Publishing.

[ref15] Moyi Okwaro F. & P.W. Geissler. 2015 ‘In/dependent collaborations: perceptions and experiences of African Scientists in transnational HIV Research’, Medical Anthropology Quarterly 29: 492–511. 25800667 10.1111/maq.12206 PMC4737198

[ref16] Nguyen V.K. 2009 ‘Government-by-exception: enrolment and experimentality in mass HIV treatment programmes in Africa’, Social Theory and Health 7: 196–217.

[ref17] Prince R.J. 2012 ‘HIV and the moral economy of survival in an East African city’, Medical Anthropology Quarterly 26: 534–56. 23361884 10.1111/maq.12006

[ref18] Prince R.J. 2013 ‘Tarmacking in the millennium city: spatial and temporal trajectories of empowerment and development in Kisumu, Kenya’, Africa 83: 582–605. 26321763 10.1017/S0001972013000478 PMC4541551

[ref19] Prince R.J. & R. Marsland, eds. 2013 Making and Unmaking Public Health in Africa: ethnographic and historical perspectives. Athens, OH: Ohio University Press.

[ref19a] Prince R.J. & H. Brown, eds. 2016 Volunteer Economies: the politics and ethics of voluntary labour in Africa. Oxford: James Currey.

[ref20] Reynolds Whyte S. & G. Etyang Siu. 2014 ‘Contingency: interpersonal and historical dependencies in HIV care’, in E. Cooper & D. Pratten, eds. Ethnographies of Uncertainty in Africa. London: Palgrave Macmillan, 19–35.

[ref21] Rottenburg R. 2009 ‘Social and public experiments and new figurations of science and politics in postcolonial Africa’, Postcolonial Studies 12: 423–40.

[ref22] Simpson B. & S. Sariola. 2012 ‘Blinding authority: randomized clinical trials and the production of global scientific knowledge in contemporary Sri Lanka’, Science, Technology and Human Values 37: 555–75.

[ref23] Stadler J.J., S. Delany & M. Mntambo. 2007 ‘Women's perceptions and experiences of HIV prevention trials in Soweto, South Africa’, Social Science and Medicine 66: 189–200. 17904718 10.1016/j.socscimed.2007.08.021

[ref23a] Sullivan N. 2012 Enacting spaces of inequality. Space and Culture 15: 57–67.

[ref24] Swidler A. & S.C. Watkins. 2009 ‘“Teach a man to fish”: the sustainability doctrine and its social consquences’, World Development 37: 1182–96. 20161458 10.1016/j.worlddev.2008.11.002 PMC2791326

[ref25] UNAIDS and the African Union Joint Report. 2015 ‘Empower young women and adolescent girls: fast-tracking the end of the AIDS epidemic in Africa’. Geneva: Joint United Nations Programme on HIV/AIDS (UNAIDS).

[ref26] Wendland C.L. 2012 ‘Moral maps and medical imaginaries: clinical tourism at Malawi's College of Medicine’, American Anthropologist 114: 108–22. 22662357 10.1111/j.1548-1433.2011.01400.x

[ref27] Whyte S.R. 1997 Questioning Misfortune: The Pragmatics of Uncertainty in Eastern Uganda. Cambridge: Cambridge University Press.

[ref28] Wright S. 1994 Anthropology of Organizations. London: Routledge.

